# The Treatment of Headaches

**Published:** 1891-10

**Authors:** Francis X. Dercum

**Affiliations:** Philadelphia


### THE TREATMENT OF HEADACHES.

BY FRANCIS X. DERCUM, M. D., OF PHILADELPHIA.

A great deal might be said in regard to the treatment of the cases that belong to the group of *migraine* In the first place, the patient should be put on such constitutional treatment as will improve the general condition and bring it up to a high standard. Every error of diet should be eliminated, not because the patient has gastric catarrh, but because he suffers with migraine. These patients should be given large amounts of milk in addition to other food. The food given should be in a digestible form. Exercise is another important factor. This should be rigidly insisted upon. However, in spite of what you do, although the attacks may be less frequent, it is improbable that you will be able to stop them altogether. I do not say that this is invariably true, but it is so in many cases. In addition, it is necessary to practice some decided interference at the time of the attack. One plan is to use morphia. Of course, that is a decided way of dealing with pain due to any cause. This is, of course, a dangerous method of dealing with pain, especially where there are recurring attacks, for the patient inevitably acquires the morphia habit. Again, especially where the migraine is associated with sympathetic spasm, the pain is often relieved by large doses of whiskey, especially if it be given hot. Various drugs have in recent times been given, not only for migraine, but also for other head pains, notably antipyrine and antifebrin. Antipyrine is a very valuable drug, and is a decided addition to our armamentarium. In some way or other the notion has got abroad, and is held by many people, that antipyrine is a dangerous drug. An ordinary individual will stand ten, fifteen, and even twenty grains without noticeable effect on the heart or pulse. Antifebrin is a dangerous drug, and should not be given in large doses. Antipyrine, however, only exceptionally acts as a depressant. A good plan is to associate the antipyrine with bromides. The depressing effect, if any, can be prevented by giving digitalis, or especially by aromatic spirits of ammonia, which is acceptable to the stomach in this condition. Cannabis indica also enjoys a reputation in the treatment of migraine, and a just one. One-fourth to one-half a grain of the extract may be given at intervals of four hours.

In the treatment of *functional headaches*, due to anæmia or chlorosis or to hyperæmic states, general principles, of course, must guide you. The anæmic patient needs the best hygiene, and especially does he need iron. He requires the most nutritious diet. The headache of chronic nervous exhaustion and neurastenia may also be spoken of here. The only way in these cases is to put the patient to bed for a while. Forced feeding, especially with milk, massage and faradization will cause improvement in the general health, and the headache will disappear. You may treat some of these cases in other ways, but you will make no impression on the headache until you put them to bed. If you make the diagnosis that the headache is due to neurasthenia, do not hesitate to tell the patient of the necessity of rest, else you will fail in relieving the headache, and your reputation will suffer.

The headaches due to disorders of nutrition, as the various *diathetic headaches*, will call for their special treatment. The uræmic, rheumatic and diabetic headaches suggest what is to be done. The same is true of the toxic headaches. I shall here refer to one point only, and that is in connection with the malarial headache. This is not, as a rule, relieved by ordinary doses of quinine, such as would prevent the recurrence of a chill. If you do not give large doses, you will fail. Twenty and even thirty grains are required to make an impression. Where it is necessary to give very large doses it is well, in order to avoid irritation of the stomach, to give the drug in ten-grain doses in capsules every hour with a quantity of milk, until the required amount is taken. In that way the quinine will dissolve slowly in the presence of the curds of the milk, and there will not be much irritation of the stomach. In order to obtain the best effect the remedy should be administered four or five hours before the expected attack.

In regard to *headaches of reflex origin*, each case will require its appropriate treatment. The eye, nasal and gastric headaches suggest their respective treatment. Gastric headaches are due to chronic gastritis, and the treatment should be directed to the relief of this condition. Do not let yourself be turned aside from the proper thing to do by dabbling with pepsin and other digestants. Put the patient on a bland diet, give him nothing but milk, perhaps with a few crackers, or stale bread. To this possibly may be added beef juice from time to time. If he will consent to give the stomach a rest he will recover. This is the most important part of the treatment. Nitrate of silver is also of service. One-fourth of a grain of nitrate of silver, with hyoscyamus or belladonna, may be given thirty minutes or an hour before meals. It should be swallowed with as small a quantity of water as possible. Even if there be formed, as some assert, an albuminate of silver, there can be no doubt that the drug has some alterative effect upon the stomach. It probably acts as a stimulant and sedative. It is probable that it rapidly undergoes chemical change, but we know that cases improve more rapidly with silver than without it. During the attack of headache it is well to give an emetic, and preferably one that will not irritate the stomach. You would not, under these circumstances, think of giving mustard. You could give the patient warm water until he could retain no more. The moment vomiting takes place the headache is relieved. The vomiting should be followed by the administration of an alkali, such as bicarbonate of soda, soda mint or a tablespoonful or two of lime water in ordinary water.

If you have an *obscure headache*, for which you can find no obvious cause, and you detect physical signs of cardiac disease, you must take this into account. Do not forget, further, the part that the bowel may play in the production of headache. Uterine headaches will require their special treatment.

*Hysterical headache* is among the most difficult to treat. Here again general principles must guide you. You must treat the hysterical patient as a neurasthenic patient. It so happens that in hysteria we almost always have neurasthenic symptoms. In order to show you the role that neurasthenia may play in hysteria, I will cite a case from my own experience. I had under my care, some years ago, a young woman who was undoubtedly hysterical. Otherwise she was apparently in good health. She had a good color; she was not anæmic. She was rather a plump woman of about twenty years, but certainly hysterical. She was placed in bed and upon the rest treatment, but, owing to the interference of the mother, it was not carried out as well as it might have been. Instead of improving she got worse, and after a time typical hemi-anæsthesia appeared. With this there appeared some thickening of the epithelium on the affected side. Tehn the fine hair which is found on the arms and legs became exceedingly brittle, so that in sweeping the hand against the hairs, they would break off. In addition, she finally developed keratitis, and eventually lost the sight of the eye upon the anæsthetic side. I merely cite this case to show how profound hysteria may be, and how marked the neurasthenia which frequently accompanies hysteria may be.—*University Medical Magazine, April, 1891.*

---

Dr. N. M. Gray, of Allegheny, Pa., says: I have tried PAPINE in two cases, and with the best effects. Both were cases of children from one to three years, and both so complicated with cerebral trouble that I feared to use opium or any of its preparations, and yet I wished for an anodyne to control some very marked symptoms. So I tried the PAPINE, and am happy to say that it had the desired effect, without any of the unpleasant consequences so often following the use of the drug in any form I have heretofore used. I think it an excellent preparation for that class of diseases, and intend to use it hereafter.

